# Development of chimeric and bifunctional antagonists for CLR/RAMP receptors

**DOI:** 10.1371/journal.pone.0216996

**Published:** 2019-05-31

**Authors:** Chia Lin Chang, Sheau Yu Teddy Hsu

**Affiliations:** 1 Department of Obstetrics and Gynecology, Chang Gung Memorial Hospital Linkou Medical Center, Chang Gung University, Kweishan, Taoyuan, Taiwan; 2 Adepthera LLC, San Jose, CA, United States of America; Duke University School of Medicine, UNITED STATES

## Abstract

CGRP, adrenomedullin (ADM), and adrenomedullin 2 (ADM2) family peptides are important neuropeptides and hormones for the regulation of neurotransmission, vasotone, cardiovascular morphogenesis, vascular integrity, and feto‒placental development. These peptides signal through CLR/RAMP1, 2 and 3 receptor complexes. CLR/RAMP1, or CGRP receptor, antagonists have been developed for the treatment of migraine headache and osteoarthritis pain; whereas CLR/RAMP2, or ADM receptor, antagonists are being developed for the treatment of tumor growth/metastasis. Based on the finding that an acylated chimeric ADM/ADM2 analog potently stimulates CLR/RAMP1 and 2 signaling, we hypothesized that the binding domain of this analog could have potent inhibitory activity on CLR/RAMP receptors. Consistent with this hypothesis, we showed that acylated truncated ADM/ADM2 analogs of 27–31 residues exhibit potent antagonistic activity toward CLR/RAMP1 and 2. On the other hand, nonacylated analogs have minimal activity. Further truncation at the junctional region of these chimeric analogs led to the generation of CLR/RAMP1-selective antagonists. A 17-amino-acid analog (Antagonist 2–4) showed 100-fold selectivity for CLR/RAMP1 and was >100-fold more potent than the classic CGRP receptor antagonist CGRP8-37. In addition, we showed (1) a lysine residue in the Antagonist 2–4 is important for enhancing the antagonistic activity, (2) an analog consisted of an ADM sequence motif and a 12-amino-acid binding domain of CGRP exhibits potent CLR/RAMP1-inhibitory activity, and (3) a chimeric analog consisted of a somatostatin analog and an ADM antagonist exhibits dual activities on somatostatin and CLR/RAMP receptors. Because the blockage of CLR/RAMP signaling prevents migraine pain and suppresses tumor growth/metastasis, further studies of these analogs, which presumably have better access to the tumor microenvironment and nerve endings at the trigeminal ganglion and synovial joints as compared to antibody-based therapies, may lead to the development of better anti-CGRP therapy and alternative antiangiogenesis therapy. Likewise, the use of bifunctional somatostatin-ADM antagonist analogs could be a promising strategy for the treatment of high-grade neuroendocrine tumors by targeting an antiangiogenesis agent to the neuroendocrine tumor microenvironment.

## Introduction

CLR/RAMP1, 2 and 3 complexes are cognate receptors for four peptides hormones, including α- and β-calcitonin gene-related peptides (α- and β-CGRPs), adrenomedullin (ADM), and adrenomedullin 2 (ADM2, or intermedin [IMD]) [1–5]. The CLR/RAMP receptor complexes contain two transmembrane components, the calcitonin receptor-like receptor (CLR) and one of the three receptor activity-modifying proteins (RAMP1, 2, and 3) [3–7]. Whereas CGRPs mainly act through the CLR/RAMP1 receptor, ADM has high affinity for CLR/RAMP2 and 3 receptors [6, 8]. On the other hand, ADM2 is a weak ligand and exhibits no distinct preference for the three CLR/RAMP receptors. Earlier studies have shown that ADM plays critical roles in the regulation of cardiovascular development, vasotone, endothelial barrier integrity, and tumor angiogenesis [3, 9–29]. Likewise, ADM2 is important for the regulation of vascular lumen enlargement, and exerts vaso- and cardio-protective effects in animals with hypertension, heart failure, ischemia reperfusion injury, obesity, or insulin resistance [30–33]. By contrast, CGRPs are important for the regulation of nociception, hyperalgesia, and allodynia [34–37].

Excessive release of CGRP is associated with the development of migraine headache, osteoarthritis pain, complex regional pain syndrome, and diabetic neuropathy [38, 39]; whereas ADM signaling is associated with tumor growth/metastasis. As such, CLR/RAMP receptor antagonists have been developed for the treatment of pain and tumor growth. Four distinct approaches have been used to block CLR/RAMP signaling: (1) peptide antagonists (e.g., CGRP8-37 and ADM22-52) [40–44], (2) small molecule antagonists (e.g., telcagepant for CLR/RAMP1) [25, 45, 46], (3) anti-CGRP or anti-ADM antibodies (e.g., galcanezumab and fremanezumab) [29, 47–50], and (4) anti-CLR or anti-RAMP antibodies (e.g., erenumab) [29, 48–51]. Although several small molecule CGRP antagonists (e.g., telcagepant) are effective in reducing migraine headache, most of them suffered concerns of liver toxicity [52]. By contrast, anti-CGRP and anti-RAMP1 antibodies have been approved as anti-migraine therapies in 2018 [36, 39, 51–56]. On the other hand, because blockage of ADM signaling suppresses tumor xenograft growth and metastasis in animals [26, 29, 43, 47, 49, 57], ADM antagonists are being developed as anti-tumor/angiogenesis therapy [26, 29, 43, 47, 49, 57, 58].

Although anti-CGRP antibody therapies showed efficacy in patients, they are inadequate for the control of severe migraine in many patients and are ineffective for reducing osteoarthritis pain [59–61]. Therefore, there is still a substantial unmet medical need of therapeutics that can better control CLR/RAMP-mediated pain response and tumor growth/angiogenesis. Because peptide antagonists have a volume of distribution ~3 times that of a typical antibody, they have better access to target receptors at the nerve endings and the tumor microenvironment. Therefore, peptide antagonists may represent alternative candidates for the development of anti-CGRP and anti-ADM therapies.

Recently, we have discovered that an acylated chimeric ADM/ADM2 analog exhibits potent agonistic activity for CLR/RAMP1 and 2. Based on this finding, we hypothesized that the binding domain of this chimeric analog could be a useful building block to develop novel CLR/RAMP receptor antagonists. In addition, because N-terminal acylation, benzoylation, or dibenzoylation of CGRP8-37 improves the affinity toward CGRP receptor [62], we further hypothesized that acylation modification may improve the antagonistic activity of chimeric analogs. Accordingly, we analyzed a series of acylated truncated ADM/ADM2 analogs. Consistent with our hypothesis, several of these chimeric analogs exhibit potent pan-specific or CLR/RAMP1-selectvie antagonistic activities. In addition, analysis of a chimeric analog consisted of a somatostatin analog and an ADM antagonist motif showed the analog exhibits potent somatostatin receptor-activation and CLR/RAMP receptor-inhibitory activities. As such, this new class of antagonistic analogs could be useful for the development of alternative anti-CGRP and novel targeted antiangiogenesis therapeutics.

## Materials and methods

### Materials

ADM, CGRP, CGRP8-37, ADM22–52 and chimeric analogs were synthesized using solid-phase peptide synthesis methodologies and obtained from Genscript Inc., Lifetein, or Karebay Inc. The synthesized product was purified by analytical RP-HPLC to >95% purity. The identity of the purified products was confirmed by MS spectrometry.

### Design of CLR/RAMP1 and 2 signaling assays

The bioactivity of synthetic analogs was studied using cells that stably express CLR/RAMP1 (1321N1 cells) or CLR/RAMP2 (CHO-K1 cells) receptors using CLR/RAMP1 cAMP and CLR/RAMP2 arrestin assays from DiscoveRx (Fremont, Ca). In receptor-activation assays, the dose-dependent stimulatory response was studied in duplicate, at 10 different concentrations. Half maximal effective concentration (EC_50_) and half maximal inhibitory concentration (IC_50_) were performed using 10-point dose response curves with a starting concentration of 1.0 or 10 μM and serially diluted 3-fold, in DMSO. Human β-CGRP was used as a positive control in the CLR/RAMP1 assay, and ADM was used as a positive control in the CLR/RAMP2 assay.

### Assay of CLR/RAMP1 signaling

For the analysis of signaling in CLR/RAMP1-expressing cells, cAMP Hunter cell lines were expanded from freezer stocks [63], and cells were seeded in white walled, 384-well microplates and incubated at 37C for the appropriate time. The activity was determined using the DiscoveRx HitHunter cAMP XS+ assay. Media was aspirated from cells and replaced with 15 μl 2:1 HBSS/10mM Hepes:cAMP XS+ Ab reagent. Intermediate dilution of sample stocks was performed to generate 4X sample in assay buffer, and 5 μl of 4X sample was added to cells and incubated at 37C or room temperature for the appropriate time. Vehicle concentration was 1%.

For the determination of antagonistic activity, cells were pre-incubated with sample followed by agonist challenge at the EC_80_ concentration. Known antagonists, including BIBN4096BS, CGRP8-37, and ADM22-52 were used as controls. Media was aspirated from cells and replaced with 10 μl 1:1 HBSS/Hepes:cAMP XS+ Ab reagent, and 5 μl of 4X compound was added to the cells and incubated at 37C or room temperature for 30 minutes. Then, 5 μl of 4X EC_80_ agonist was added to cells and incubated at 37C or room temperature for the appropriate time.

After compound incubation, assay signal was generated through incubation with 20 μl cAMP XS+ ED/CL lysis cocktail for 1 hr followed by incubation with 20 μl cAMP XS+ EA reagent for 3 hr at room temperature. Microplates were read with a PerkinElmer instrument for chemiluminescent signal detection. The compound activity was analyzed using a CBIS data analysis suite (ChemInnovation, CA). For agonist mode assays, percentage activity was calculated using the following formula: % Activity = 100% x (mean RLU of test sample—mean RLU of vehicle control)/(mean MAX control ligand—mean RLU of vehicle control). For antagonistic activity assays, percentage inhibition was calculated using the following formula: % Inhibition = 100% x (1 - (mean RLU of test sample—mean RLU of vehicle control)/(mean RLU of EC_80_ control—mean RLU of vehicle control)).

### Assay of CLR/RAMP2 receptor signaling

The CLR/RAMP2 signaling was assayed using the CLR/RAMP2 PathHunter β-Arrestin assay [64]. In this assay, the GPCR was fused in frame with a small enzyme donor fragment ProLink (PK) and co-expressed in cells stably expressing a fusion protein of β-arrestin and an N-terminal deletion mutant of β-galactosidase (i.e., enzyme acceptor or EA). Activation of the CLR/RAMP2 stimulates binding of β-arrestin to the PK-tagged receptor and leads to an increase in enzyme activity that can be measured using chemiluminescent PathHunter Detection Reagents. PathHunter cell lines were seeded in white walled, 384-well microplates and incubated at 37C prior to testing. For agonist determination, intermediate dilution of sample stocks was performed to generate 5X sample in assay buffer, and 5 μl of 5X sample was added to cells and incubated at 37C for 90 minutes. Vehicle concentration was 1%. For antagonistic activity determination, cells were pre-incubated with antagonist followed by agonist challenge at the EC_80_ concentration. Assay signal was generated through a single addition of 12.5 or 15 μl (50% v/v) of PathHunter Detection reagent cocktail, followed by 1 hr incubation at room temperature.

### Assay of somatostatin receptor 2 (SSTR2) signaling

The effect of somatostatin-related peptides on somatostatin receptor 2 (SSTR2) signaling was assayed using the cAMP Hunter CHO-K1 SSTR2 Assay (DiscoveRx Inc.). Cells overexpressing SSTR2 were cultured and assayed using the agonistic mode as described for the study of CLR/RAMP1 receptor signaling. The functional status of the receptor was monitored by measuring the cellular cAMP levels using a gain-of-signal competitive immunoassay based on a β-galactosidase enzyme fragment complementation method.

## Results

### Design of CLR/RAMP receptor agonists and antagonists

**Fig 1:**
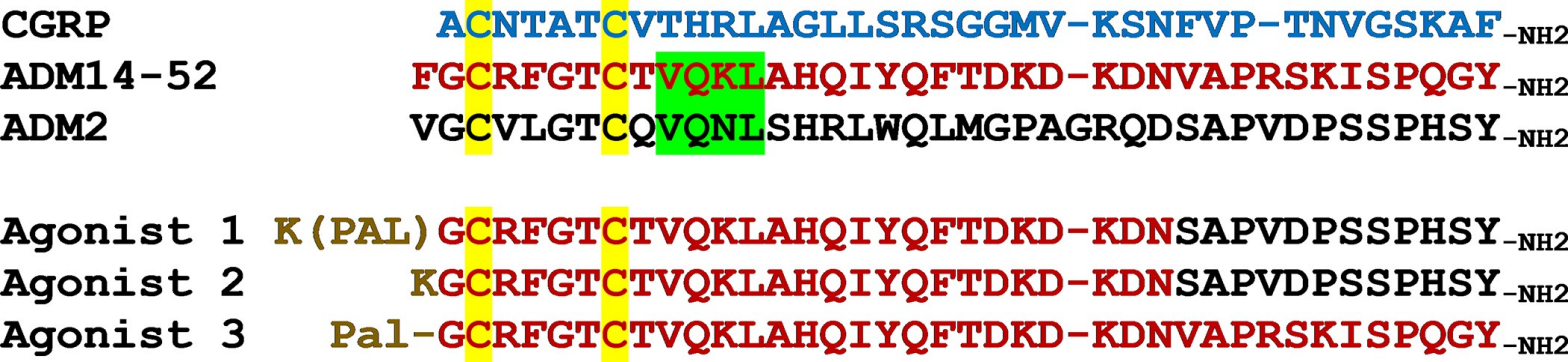
Sequence alignment of CLR/RAMP receptor agonists. The sequence alignment includes CGRP (blue letters), adrenomedullin 14–52 (ADM14-52; red letters), adrenomedullin 2 (ADM2; black letters), as well as Agonists 1, 2 and 3. The N-terminal cysteines that form a disulfide ring are indicated by a yellow background. The region that is critical for the derivation of truncated ADM/ADM2 antagonists is indicated by a green background. The origin of individual residues in chimeric analogs is indicated by the color of residues. The N-terminal modifications, including palmitoylation (Pal) and lysine-conjugated palmitoylation (Pal-K or K(pal)), are indicated by brown letters.

**Fig 2:**
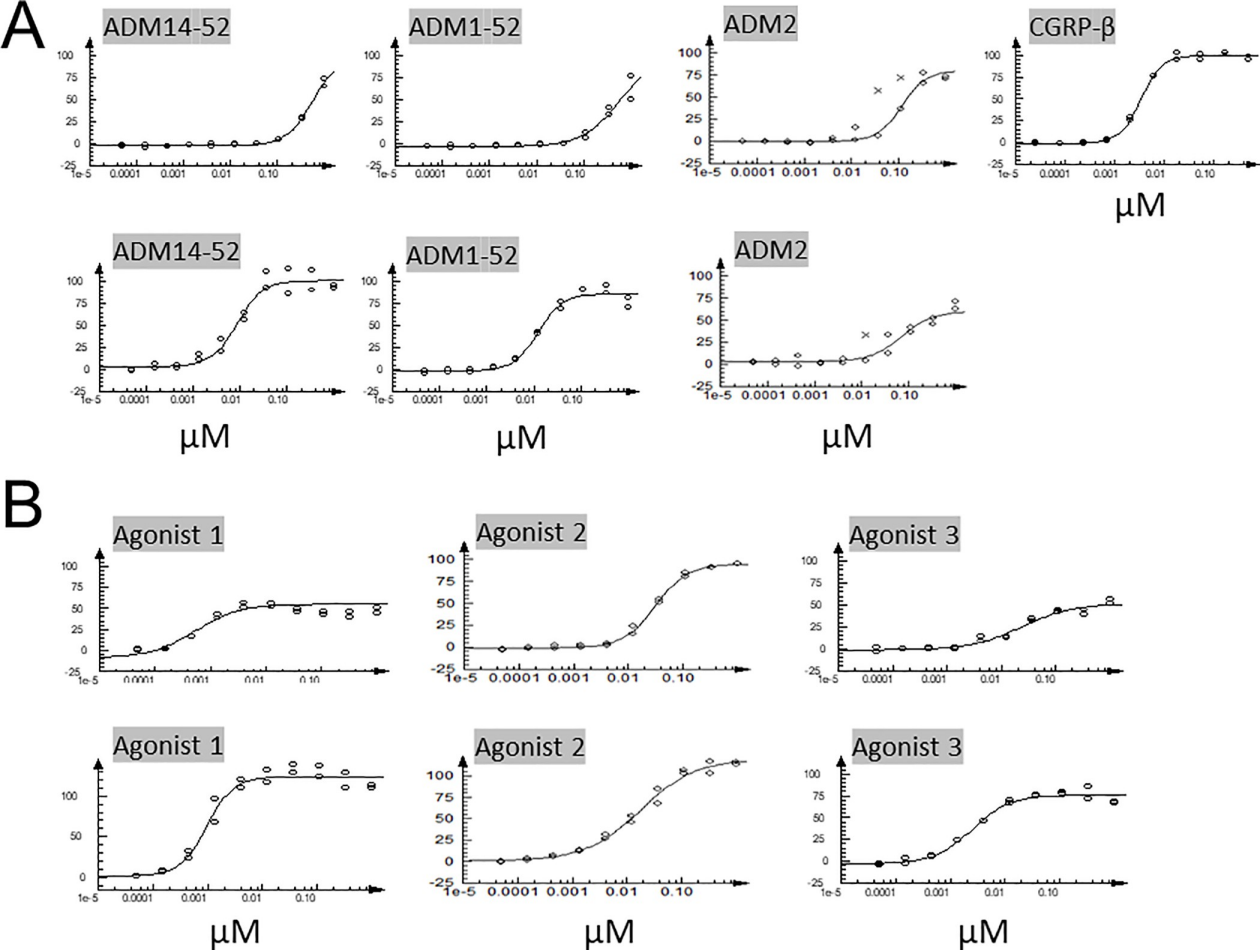
Dose-response curves of chimeric agonists. The stimulatory effects of positive controls (i.e., ADM14-52, ADM1-52, ADM2, and CGRP-β)(A) and Agonists 1, 2 and 3 (B) on CLR/RAMP1 (upper panel) and 2 (lower panel) signaling are presented as dose-response curves. CGRP-β is a strict CLR/RAMP1 receptor agonist; only the effect on CLR/RAMP1 signaling is presented.

In an effort to characterize the interaction of ADM2 with CLR/RAMP receptors, we found that an acylated chimeric ADM/ADM2 analog (Agonist 1, Fig 1) potently stimulates CLR/RAMP1 and 2 signaling (Table 1). The receptor-activation activity of this analog was distinctly different from those of wild-type CGRP, ADM, and ADM2, which are not acylated (Table 1; Fig 2). The EC_50_ of wild-type ADM (i.e., ADM1-52 or ADM14-52) for CLR/RAMP2 is ~9–12 nM; whereas the EC_50_ for ADM2 was 70 nM (Fig 2A). ADM and ADM2 had low potencies on the activation of CLR/RAMP1 with EC_50_ values >100 nM. On the other hand, CGRP had an EC_50_ of 1.1–3.4 nM for CLR/RAMP1. By contrast, the EC_50_ values for activating CLR/RAMP1 and 2 by the chimeric Agonist 1 was ~0.5 and 1 nM, respectively (Fig 2B). By contrast, the EC_50_ of a corresponding analog without an acylation modification (Agonist 2) was 31 and 18 nM for CLR/RAMP1 and 2, respectively. Acylation modification of a wild-type ADM also increased the potency of the ADM analog (Agonist 3), but to a limited extent.

**Table 1:** Bioactivity of synthetic CLR/RAMP1 and 2 receptor agonists.

Identity	CLR/RAMP1		CLR/RAMP2	
	EC_50_ (nM)	Max Activity	EC_50_ (nM)	Max Activity
		% of control		% of control
**Wild-type ADM, ADM2, and CGRP**				
ADM14-52	540	69	9	102
ADM1-52	564	63	12	91
ADM2	116	72	70	67
CGRP	1.1–3.4	103		
**Modified agonistic peptides**				
Agonist 1	0.5	48	1	119
Agonist 2	31	95	18	115
Agonist 3	24	53	3	78

The agonistic activity is described as EC_50_ and the maximum activity in % of a positive control. The positive controls for CLR/RAMP1 and 2 signaling are CGRP and ADM, respectively.

**Fig 3:**
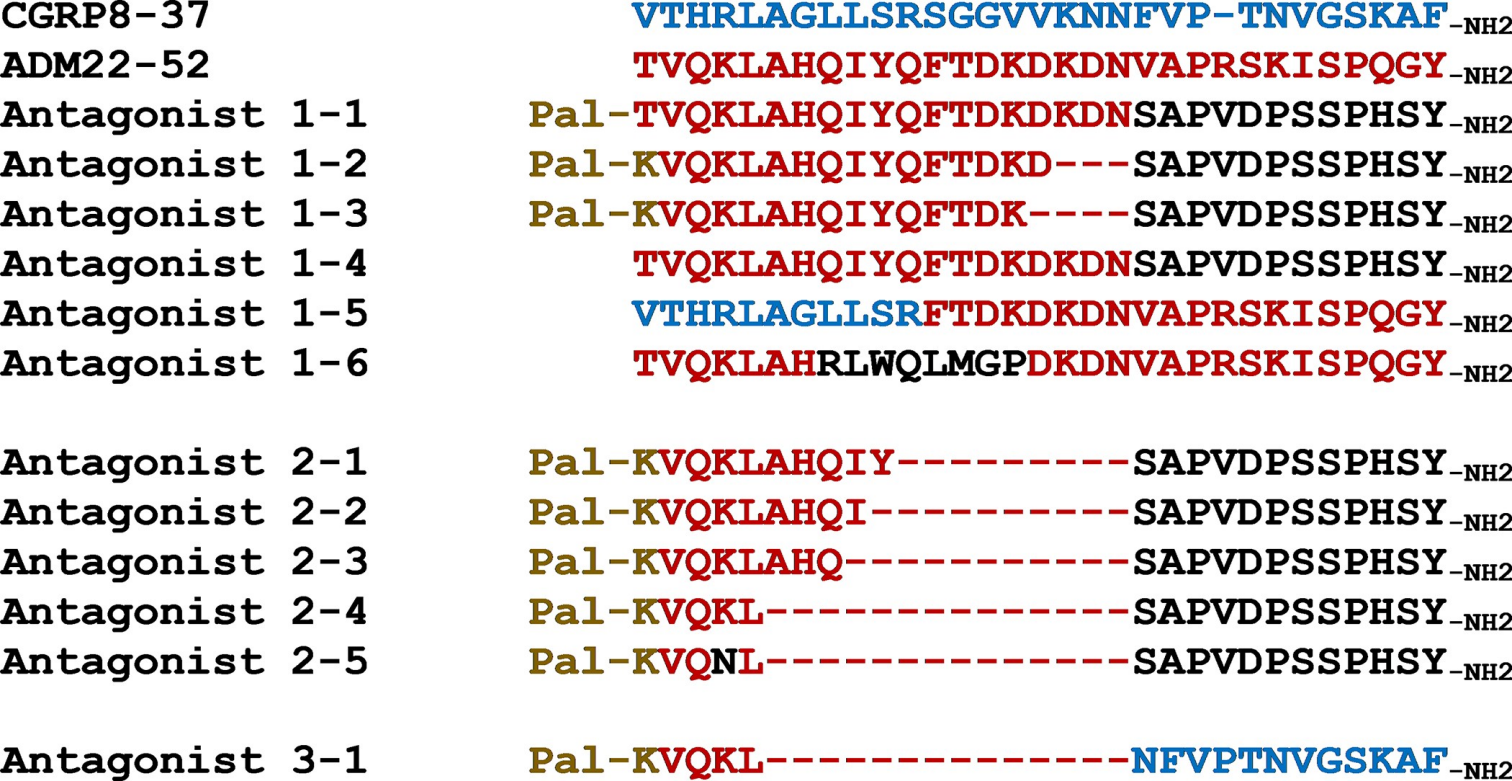
Sequence alignment of chimeric antagonists. The sequence alignment includes CGRP8-37 (blue letters), ADM22-52 (red letters), Antagonists 1–1 to 1–6, Antagonists 2–1 to 2–5, and Antagonist 3–1. The origin of individual residues in chimeric analogs is indicated by the color of residues. The N-terminal modifications, including palmitoylation (Pal) and lysine-conjugated palmitoylation (Pal-K), are indicated by brown letters. Sequence gaps are indicated by dash lines.

Because the activity of CGRP/ADM/ADM2 family peptides can be partly attributed to the degree of interaction between the C-terminal binding domain and the receptor extracellular domain (ECD) [65], we hypothesized that the binding domain of Agonist 1 could possess unique antagonistic activity toward CLR/RAMP receptors. In addition, because conjugation of a hydrophobic moiety at the N-terminus of CGRP8-37 improves receptor-interacting affinity, we appended a palmitic acid at the N-terminus of various chimeric analogs (Fig 3).

### Truncated chimeric ADM/ADM2 analogs potently inhibit CLR/RAMP1 and/or 2 signaling

**Fig 4:**
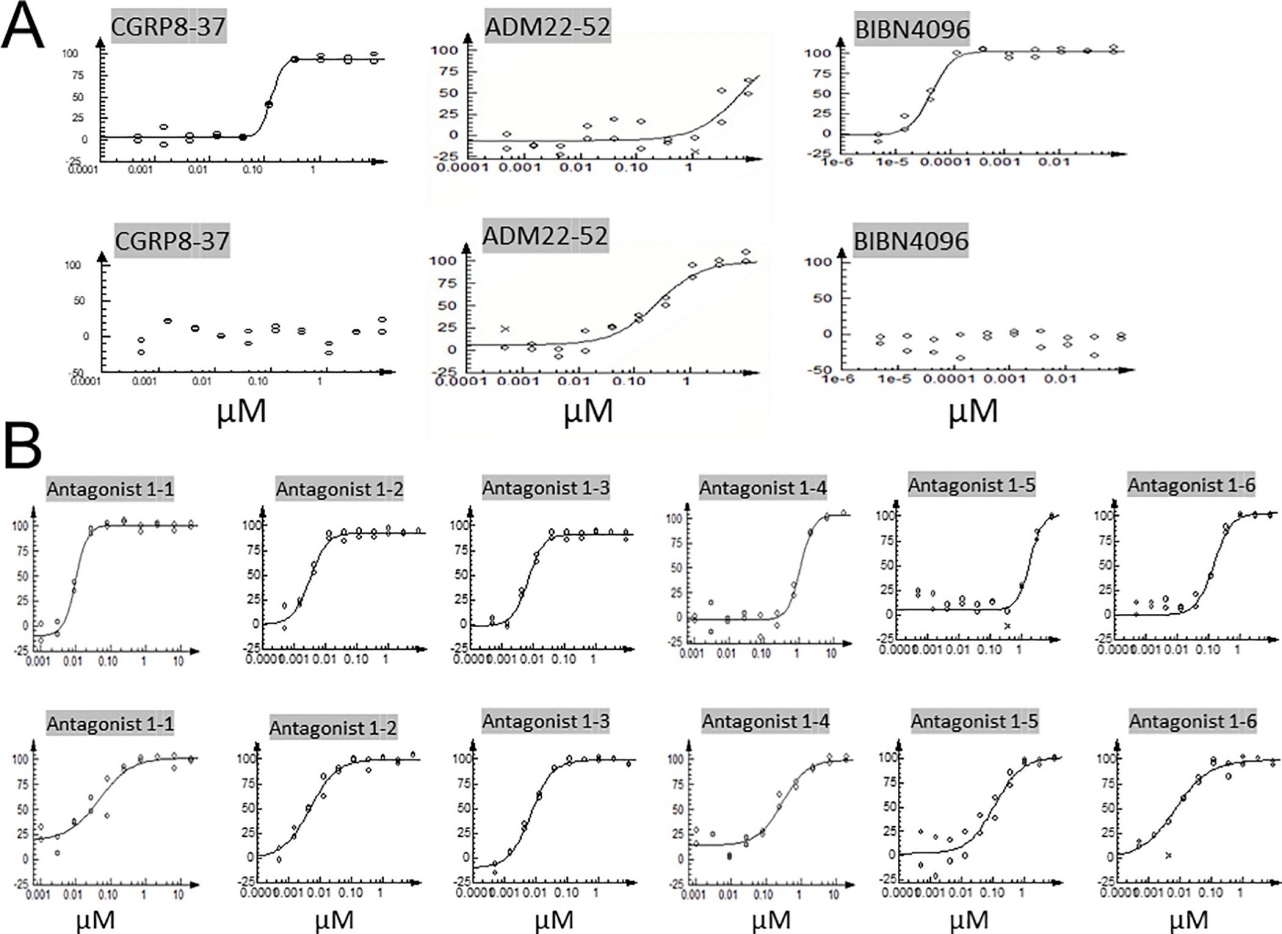
Dose-response curves of chimeric antagonists. The inhibitory effects of positive controls (i.e., CGRP8-37, ADM22-52, and BIBN4096) (A) and Antagonists 1–1 to 1–6 (B) on CLR/RAMP1 (upper panel) and 2 (lower panel) signaling are presented as dose-response curves in the presence of an EC_80_ dose of CGRP or ADM, respectively.

CGRP8-37 and ADM22-52 are classic antagonists that exhibit strict preference for CLR/RAMP1 and 2, respectively (Fig 2). Analysis of receptor signaling at the antagonistic mode showed that ADM22-52 inhibits CGRP-mediated CLR/RAMP1 and ADM-stimulated CLR/RAMP2 signaling with IC_50_ values of 6600 and 256 nM, respectively (Table 2, Fig 4A). On the other hand, CGRP8-37 had IC_50_ values of 133 and >10000 nM, for CLR/RAMP1 and 2, respectively.

**Table 2:** Antagonistic activity of chimeric CLR/RAMP receptor antagonists.

Identity	CLR/RAMP1		CLR/RAMP2	
	IC_50_ (nM)	Max Activity	IC_50_ (nM)	Max Activity
		% of control		% of control
**BIBN4096**	0.05	105	>100	0
**Wild-type peptides**				
ADM22-52	6600	57	256	105
CGRP8-37	133	95	>10,000	15
**Pan-specific chimeric antagonists**				
Antagonist 1–1	9.9	101	47	100
Antagonist 1–2	3.2	94	4.9	104
Antagonist 1–3	7	93	7.1	100
**Low-potency chimeric antagonists**				
Antagonist 1–4	1123	106	289	101
Antagonist 1–5	1878	99	117	100
Antagonist 1–6	152	101	7.3	101

The antagonistic activity on CGRP-mediated CLR/RAMP1 and ADM-mediated CLR/RAMP2 signaling is described as IC_50_ and the maximum activity in % of a positive control. The potency of a small molecule CGRP antagonist, BIBN4096, is provided for comparison.

Analysis of an acylated 31-amino–acid ADM/ADM2 chimera (Antagonist 1–1, Table 2) and analogs with additional deletion at the junctional region of Antagonist 1–1 (i.e., Antagonists 1–2 [28 residues] and 1–3 [27 residues]) showed these chimeras exhibit potent antagonistic activity for both CLR/RAMP1 and 2 (Fig 4B). The IC_50_ values of these chimeras for CLR/RAMP1 were 10-fold lower than that of CGRP8-37. Likewise, the IC_50_ values for CLR/RAMP2 were 5- to 50-fold lower than that of ADM22-52. By contrast, a nonacylated analog of Antagonist 1–1 (Antagonist 1–4), a CGRP/ADM chimera (Antagonist 1–5), and an ADM/ADM2/ADM chimera (Antagonist 1–6) showed lower bioactivity when compared with acylated ADM/ADM2 chimeras.

### An ADM-derived motif is important for enhancing the antagonistic activity of chimeric analogs

**Fig 5:**
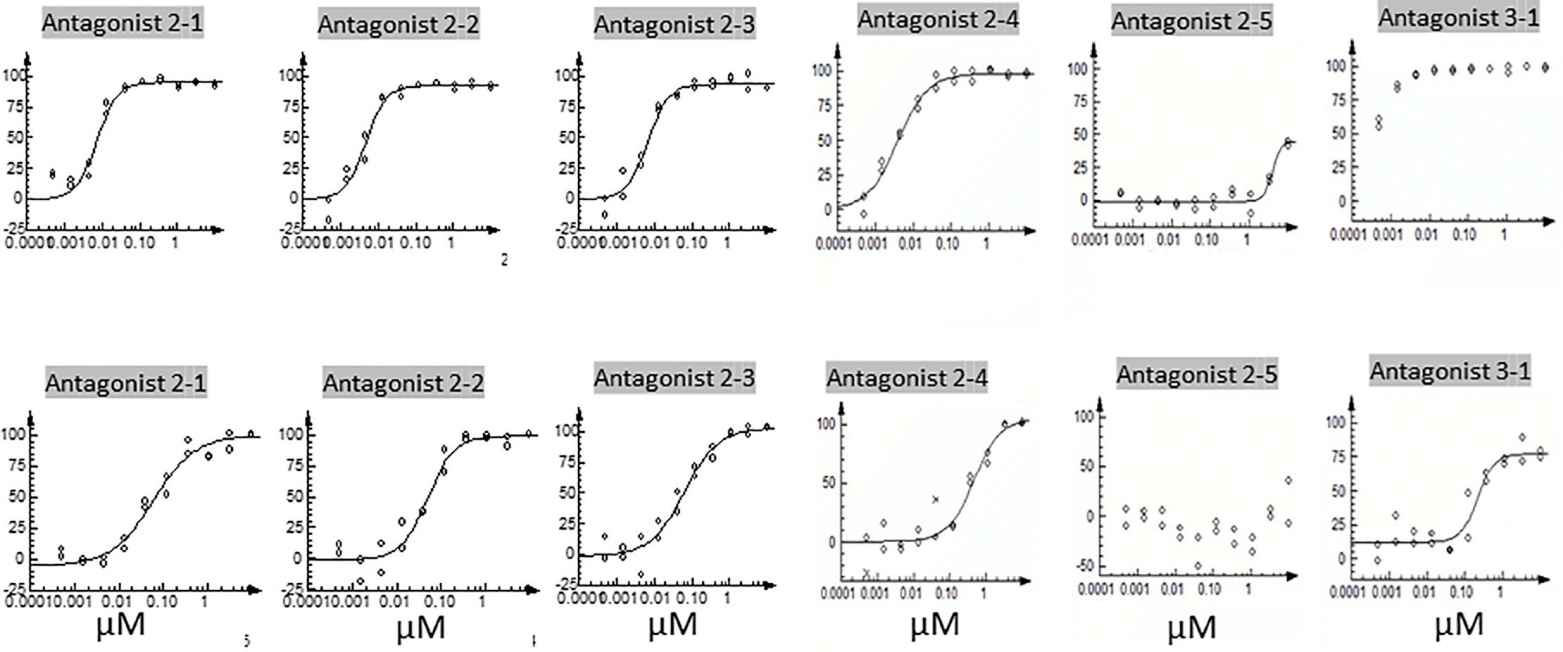
Dose-response curves of miniaturized antagonists. The inhibitory effects of Antagonists 2–1 to 2–5, and 3-1on CLR/RAMP1 (upper panel) and 2 (lower panel) signaling are presented as dose-response curves in the presence of an EC_80_ dose of CGRP or ADM, respectively.

To determine whether the enhanced activity of chimeric antagonists is affected by additional residue deletion, we studied analogs that contain further truncation at the junctional region of chimeric analogs (i.e., Antagonists 2–1 to 2–4 [17–22 residues]; Fig 3). These additional truncations had minimal effects on the antagonistic activity toward CLR/RAMP1 but reduced the antagonistic activity toward CLR/RAMP2 when compared to Antagonists 1–2 and 1–3 (Table 3, Fig 5). As such, these short analogs represent CLR/RAMP1-selective antagonists.

**Table 3:** Antagonistic activity of miniaturized CLR/RAMP receptor antagonists.

Identity	CLR/RAMP1		CLR/RAMP2	
	IC_50_ (nM)	Max Activity	IC_50_ (nM)	Max Activity
		% of control		% of control
**BIBN4096**	0.05	105	>100	0
**Wild-type peptides**				
ADM22-52	6600	57	256	105
CGRP8-37	133	95	>10,000	15
**Chimeric ADM/ADM2 analogs**				
Antagonist 2–1	7.3	95	61	100
Antagonist 2–2	4.7	94	50	101
Antagonist 2–3	6.7	95	64	103
Antagonist 2–4	3.8	98	462	101
Antagonist 2–5	3837	43	>10,000	14
**Chimeric ADM/CGRP analog**				
Antagonist 3–1	<0.5	100	214	80

The antagonistic activity on CGRP-mediated CLR/RAMP1 and ADM-mediated CLR/RAMP2 signaling is described as IC_50_ and the maximum activity in % of a positive control. The potency of a small molecule CGRP antagonist, BIBN4096, is provided for comparison.

Of interest, sequence comparison showed the N-terminal ADM sequence of the 17-amino-acid Antagonist 2–4 is only one amino acid different from the corresponding region of ADM2 (VQKL in Antagonist 2–4 vs. VQNL in ADM2; highlighted with a green background in Fig 1), suggesting this residue may play a role in shaping the bioactivity of Antagonist 2–4. Consistent with this hypothesis, substitution of the lysine residue in Antagonist 2–4 with an asparagine residue led to a 1000-fold reduction of the CLR/RAMP1-inhibitory activity (i.e., Antagonist 2–5).

In addition, studies of an ADM/CGRP chimera that contains an N-terminal ADM motif and a C-terminal 12-amino-acid fragment of CGRP (i.e., CGRP26-37; Antagonist 3–1, Table 3), which was known to have minimal bioactivity, showed this chimera has an IC_50_ at the subnanomolar range for CLR/RAMP1, and an IC_50_ that is >200 nM for CLR/RAMP2.

### Chimeric unimolecular somatostatin-ADM antagonist analog exhibits dual activities on somatostatin and CLR/RAMP receptors

**Fig 6:**
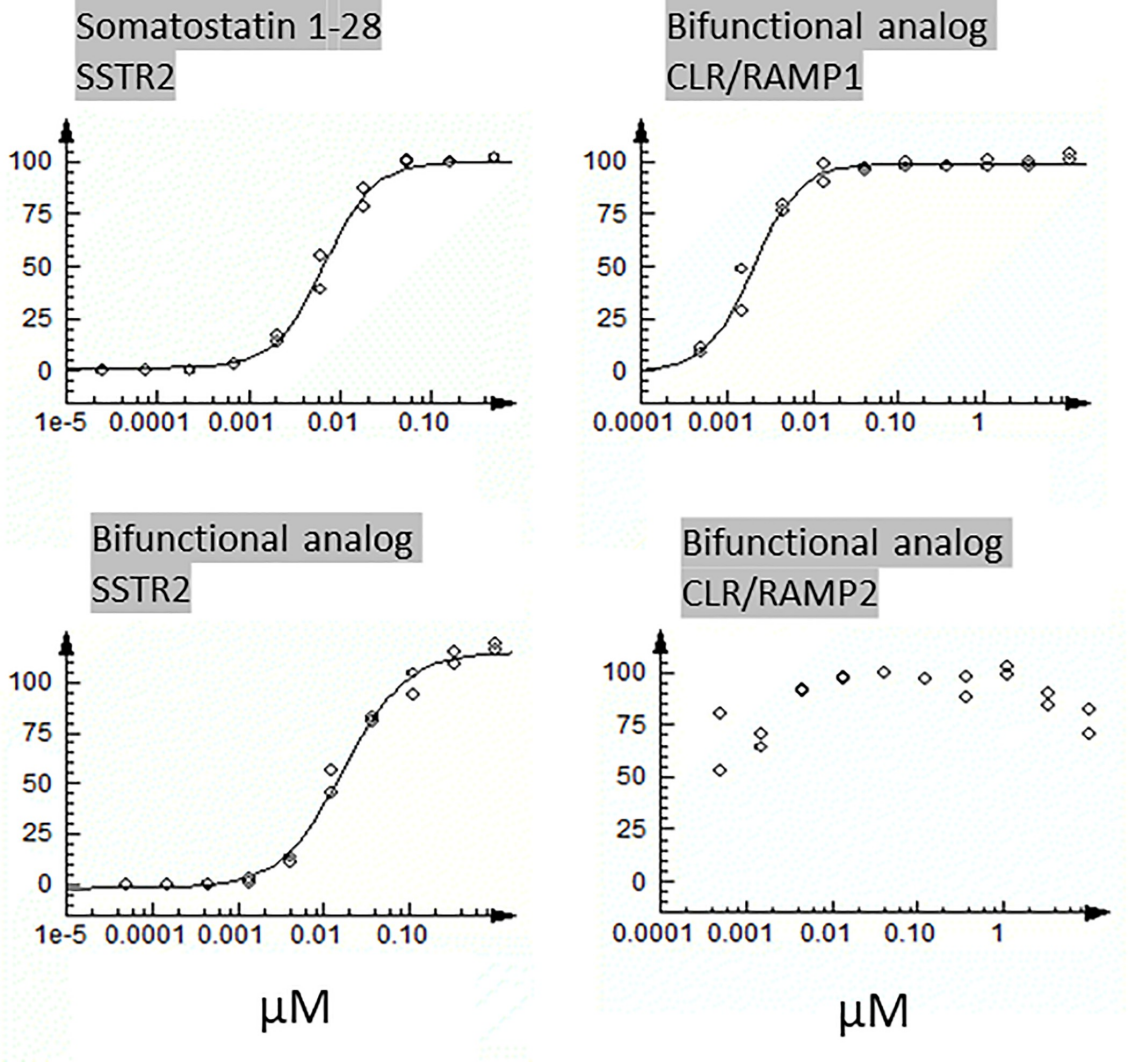
Dose-response curves of a bifunctional octreotide-ADM antagonist analog. The stimulatory effects of somatostatin1-28 and the bifunctional analog on somatostatin receptor 2 (SSTR2) signaling (left panel), and the inhibitory effects of the bifunctional analog on CLR/RAMP1 and 2 signaling (right panel) are presented as dose-response curves.

Because a targeted molecule could provide more specific therapeutic activity, ADM antagonists that contain a somatostatin receptor-interacting motif could sequester the antagonist to the neuroendocrine tumor (NET) microenvironment and be useful for the treatment of high-grade NETs which express high levels of somatostatin receptors. Analysis of a chimeric analog that contains the somatostatin analog octreotide ((D-Phe)CF(D-Trp)KTCT) and a 28-amino-acid ADM antagonist sequence (K(Pal)VQKLAHQIYQFTDKDVAPRSKISPQGY) showed it possesses potent somatostatin receptor 2 (SSTR2)-activation activity and inhibitory activities on CLR/RAMP1 and 2 signaling (Table 4, Fig 6). The EC_50_ for activating SSTR2 is similar to that of somatostatin 1–28, and the inhibitory effects on CLR/RAMP1 and 2 signaling are at the same order as the pan-specific antagonistic analogs in Table 2.

**Table 4:** The receptor-regulatory activity of a chimeric octreotide-ADM antagonist analog.

Identity	CLR/RAMP1		CLR/RAMP2		SSTR2	
	IC50 (nM)	Max Activity	IC50 (nM)	Max Activity	EC50 (nM)	Max Activity
**CGRP8-37**	133	95	>10,000	15		
**ADM22-52**	6600	57	256	105		
**Somatostatin 1–28**					6.8	102
**Octreotide-ADM antagonist analog**	2	102	<0.5	87	17	117

## Discussion

Based on the analysis of CLR/RAMP1 and 2 signaling, we showed that (1) acylated ADM/ADM2 chimeras exhibit antagonistic activities one to two orders stronger than those of CGRP8-37 and/or ADM22-52, and (2) chimeric octreotide-ADM antagonist analog exhibits dual regulatory activities toward somatostatin and CLR/RAMP receptors. In addition, the data indicated that (1) N-terminal acylation and a lysine residue within the ADM motif of chimeric analogs are important for enhancing the antagonistic activity and (2) the sequence motif encompassing residues 22–40 of ADM is important for the interaction between chimeric ADM/ADM2 antagonists and CLR/RAMP2. Further characterization of these peptidomimetics may lead to the development of therapeutics that can better inhibit pathological CGRP and/or ADM signaling in patients.

**Fig 7:**
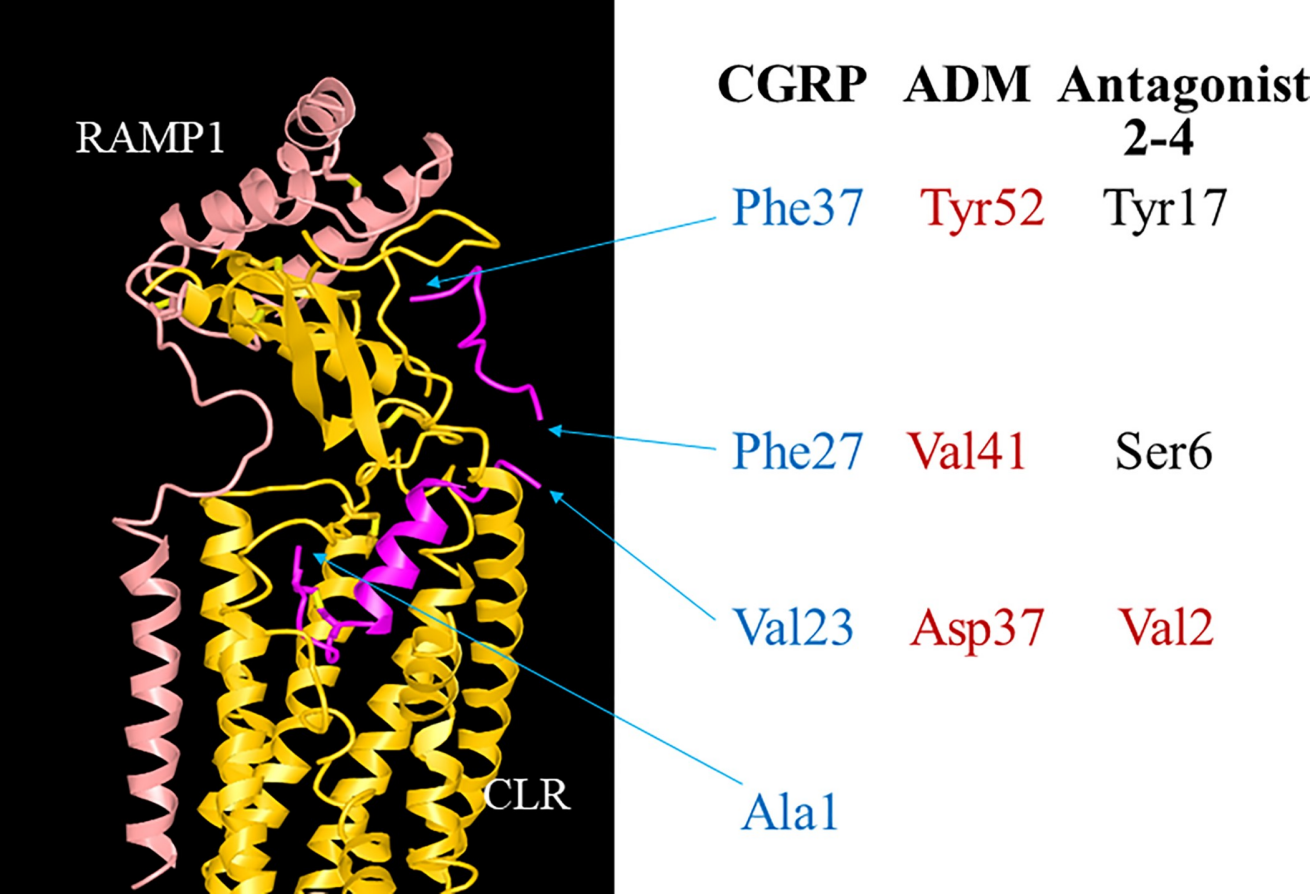
Visualization of the linker region in the CGRP peptide, which corresponds to the “junctional” region of chimeric antagonists. The interaction of chimeric antagonists with CLR/RAMP receptors could be similar to that between CGRP and the CGRP receptor complex as demonstrated by the RCSB protein data bank [PDB] structure 6E3Y [82]. The structure presented includes the CGRP (red), RAMP1 (pink) and CLR (yellow) components. The structure at the linker region of CGRP (Lys24- Asn26) was not resolved and is presented as a gap between the N- and C-terminal regions. The positions of Val23 and Phe27, which are next to the breakpoint as well as the C-terminal Phe37 of CGRP are indicated by arrows. The residues corresponding to Val23, Phe27, and Phe37 of CGRP in ADM and Antagonist 2–4 are presented next to the CGRP residues. Residues that were derived from ADM and ADM2 are indicated by red and black letters, respectively.

Similar to calcitonin and amylin, CGRP/ADM/ADM2 family peptides have an N-terminal disulfide-bond ring followed by a helix region and an unstructured C-terminal region. These ligands presumably interact with the receptors via a two-domain model in which the C-terminal region binds the receptor ectodomain, while the N-terminal region activates the receptor [66]. Earlier studies have categorized the 37-amino-acid CGRP into four distinct domains: (1) a seven-residue ring structure, (2) an α-helix composed of residues 8–18, (3) a β-bend around residues 19–27, and (4) the C-terminal binding terminus [67–70]. Whereas Thr30, Val32, Gly33, and Phe37 in the CGRP C-terminus are key residues for CLR/RAMP1 interaction, residues 19–26 help maintain the structure at the C-terminus [69–75]. The deletion of N-terminal ring domain renders the truncated CGRP8-37 peptide a competitive antagonist with a 10-fold less affinity compared to CGRP [43, 76, 77]. Additional truncation of the CGRP8-37 sequence leads to further reduction of the bioactivity [68, 78]. Similarly, the main binding epitope of ADM is located at the C-terminal 8 amino acids, and the Ile47, Gly51, and Tyr52 residues are critical for CLR/RAMP2 binding [65, 79, 80]. In addition, recent structure analyses indicated that CGRP and ADM bind a common site on CLR, and an allosteric modulation of CLR and RAMP contacts cooperates to determine CGRP and ADM selectivity [81–83]. Structural analysis also indicates that ADM2 could act via a mechanism similar to that of ADM or CGRP [66, 84]. Specifically, CGRP was shown to form extensive interactions with CLR/RAMP1 with 61.5% of the peptide surface buried (Fig 7). The N-terminus of CGRP (Ala1-Val23) tightly interacts with the receptor core, whereas the C-terminal region (Phe27-Phe37) interacts with the CLR ECD and RAMP1. On the other hand, the structure at the linker region (Lys24-Asn26) between the N- and C-terminal receptor-interacting domains was poorly resolved, perhaps due to a high mobility of this region (Fig 7, the missing linker region is represented by a gap between Val23 and Phe27) [82]. It has been suggested that this linker region could be important for enabling the N-terminus to be buried within CLR and the C-terminus to interact with CLR ECD and RAMP1. Because the junctional regions in chimeric ADM/ADM2 antagonists correspond precisely to the linker region in CGRP, the “linker region” within select chimeric antagonists may allow the analog to better interact with CLR and RAMP1 and exert potent antagonistic activities.

Earlier studies have shown that (1) benzoylated derivatives of CGRP8-37 have substantially increased binding affinities for the CGRP receptor [62], and (2) lipidated CGRP8-37 and CGRP7-37 analogs have higher bioactivity [85]. These modifications may facilitate the interaction with CLR/RAMP receptors given a key feature of the peptide-binding sites in CLR/RAMP1 is a hydrophobic patch extending from the base of CLR loop 4 to loop 3 [62, 82]. Alternatively, the hydrophobic modification may provide a better mimic of the membrane environment that a ligand encounters in association with a 7-transmembrane receptor [34, 86, 87]. Therefore, the enhanced bioactivity of chimeric antagonists could be partly attributed to these forces or conformational changes introduced by the N-terminal acylation together with the chimeric sequence.

CGRP is primarily released from C and Aδ sensory nerves, which are important for the modulation of inflammatory response, blood pressure, and auditory nerve development [34]. Excess CGRP release during neurogenic inflammation could lead to migraine headache, osteoarthritis pain, and other diseases [34, 35]. So far, four distinct approaches, including (1) peptide antagonists (e.g., CGRP8-37) [41–44], (2) small molecule antagonists (e.g., telcagepant and olcegepant) [45, 46], (3) anti-CGRP antibody [50], and (4) anti-RAMP1 antibody [50, 51] have been used to block CLR/RAMP1 signaling. Although several small molecule CGRP antagonists are effective in the treatment of migraine headache, they can lead to liver toxicity [52, 88–90]. On the other hand, several anti-CGRP/RAMP1 antibody-based therapies have been approved for the treatment of chronic migraine recently [39, 53, 54, 91–97]. However, a large fraction of migraine patients failed to respond to the anti-CGRP antibody therapies [36, 39, 51, 52, 55, 56]. Because antibody has a low volume of distribution, and the anti-CGRP antibodies mainly act by reducing the circulating level of CGRP or CGRP signaling in cells that are in close proximity of the vascular system [36, 96, 98, 99], there remains a large unmet medical need of therapies for patients with severe migraine. Therefore, potent peptide antagonists, which have better access to nerve endings and a high safety margin, may represent alternative therapeutics for better control of CGRP signaling in patients [39, 100–102].

In addition, the peptide antagonist could be useful for the treatment of osteoarthritis pain. It has been shown that CGRP and its receptor increase in synovial cells, infrapatellar fat pad, and dorsal root ganglion neurons innervating knee joints in osteoarthritis patients [103–106]. In animal models, CGRP increases acute neurogenic inflammation and joint pain [107, 108]; whereas CGRP antagonists reduces osteoarthritis pain [61, 107, 109]. However, an anti-CGRP antibody (i.e., galcanezumab) failed to reduce osteoarthritis pain in patients [59, 60]. The lack of efficacy could be due to the inability of antibodies to reduce CGRP in the synovial joint to a therapeutic level. Therefore, peptide antagonists, which have better access to nerve endings in the joints, may provide an alternative path for the development of anti-osteoarthritis pain therapy. Furthermore, because ADM signaling has been implicated in the regulation of inflammatory heat hyperalgesia and spinal glial activation [110–113], the pan-specific antagonists described here may be useful for spontaneous blockage of CGRP- and ADM-mediated pain responses.

ADM plays an important role in the regulation of angiogenesis and exhibits anti-inflammatory effects. Earlier studies have shown ADM22-52, small molecule antagonist, anti-ADM antibody, and anti-CLR/RAMP antibodies block the growth and/or metastasis of tumor xenografts in animal models [25, 26, 29, 43, 47, 49, 57, 114]. Because known peptide antagonists have low potency and short half-life, and because the antibody-based strategy has a low volume of distribution, the pan-specific antagonists may represent promising candidates for the treatment of tumor angiogenesis/metastasis and for improving tumor immune-surveillance. Among the antagonistic analogs, the bifunctional unimolecular octreotide-ADM antagonist analog could be particularly useful for the treatment of high-grade NETs. The bifunctional analog could use the NET cell’s unique characteristics (i.e., the expression of somatostatin receptors) to target the ADM antagonist to the NET microenvironment and increase tumor accumulation. As such, the bifunctional analog could have more potent anti-tumor growth/metastasis activities compared to current somatostatin analog-based therapies. It is also important to note that the bifunctional analog could represent a prime candidate for the development of a tyrosine kinase receptor-independent antiangiogenesis therapy for other cancers. For example, the bifunctional analog could be particularly useful for the treatment of castration-resistant prostate cancer. Emerging evidences have shown that (1) neuroendocrine differentiation (NED) secondary to androgen deprivation therapy (ADT) occurs frequently in metastatic castrate-resistant prostate cancer [115], and (2) somatostatin analogs increase the therapeutic window of ADT in patients with castration-resistant prostate cancer [116, 117].
